# Mess. Scott and Taynton, on Cold Water in Gout

**Published:** 1805-02-01

**Authors:** James Scott, R. T. Taynton

**Affiliations:** Bromley, Kent; Bromley, Kent


### *To the Editors of the Medical and Physical Journal.*

GENTLEMEN,

THE opinion of the medical world being at present much divided, on the propriety of applying cold water in the cure of gouty inflammation; we feel it incumbent on us to give as much publicity as possible to the following case, as it appears to us attended with peculiar circumstances.

Mr. Mace, a gentleman residing at Chislehurst in this county, about forty years of age, who had been subject for many years to severe fits of the gout, sent for us to visit him on the 10th of October, 1803. We found him complaining complaining of general indisposition, with one ancle much inflamed, swoln, and exquisitely painful. Cloths wetted in a cold solution were wrapped round the part affected, and a cordial medicine was administered internally. On the 11th, the pain and inflammation in the ancle were lessened; and the knees were now become painful: the same application was made to them. On the 12th, at night, he felt severe pain in the head, and great uneasiness in the stomach, attended with nausea and flatulence. A draught with ether and laudanum was ordered him. On the 13th he was better in every respect; the head and stomach relieved, but the inflammation in the extremities *had not returned*; 14th, continued mending; 15th, free from all pain; 20th, able to walk, without any weakness in either feet or knees.

He remained in perfect health for twelve months. About the middle of October, 1804, he had an attack similar to the one of the preceding year. The right ancle was the part chiefly affected. He was treated in the same manner as before; and was able in a few days, to pursue his usual avocation. But, on the 16th of November, we were much disappointed to find him again suffering severe pain and inflammation in the same joint. He is a sensible, intelligent man, and had paid great attention to the progress and symptoms of the disease, under this new mode of treatment. He now informed us, that he thought the gout had lurked in his habit ever since the last attack. At the same time, he felt convinced, that though the application of cold might not eradicate the disorder, yet it afforded him so much ease, that he was extremely anxious to make another trial of it. To this we readily assented. After having diligently pursued this plan till the 22d, he desisted, as nothing apparently but weakness remained. On Monday, November 26th, the *left* ancle became inflamed and painful. Without the least hesitation, he had immediate recourse to his favourite remedy; but he had no sooner applied the first wet cloth, than he felt an unusual torpor pervade his whole frame. He suffered intense pain in the stomach, attended with frequent vomiting: the head was much affected, and he was at intervals delirious. He took hot brandy and water, and heated wine with spice, which afforded him some relief. At the same time the cold was discontinued, which had been used for about two hours. On the 27th he was better, though very languid. The inflammation *did not return in*

*the*

*the left ancle.* On the 28th he continued tolerably well. But, on the 29th, a more violent attack than any of the former took place in the right hand. It is to be remarked, that on this day, though the hand was enveloped in flannel, the stomach, head, and constitution at large, were as much, if not more affected, than on the Monday when the cold was applied. He passed a most dreadful night on Thursday; and, on Friday morning, the symptoms of disorder in the head and stomach having abated, while the pain and inflammation of the hand remained undiminished, we *again* determined to apply cold, carefully watching the effect. This treatment now was attended with the happiest consequences: he experienced almost immediate ease; no other untoward symptom occurred, and he was soon restored to health.

We forbear making any comments on the above facts; but it appears worthy of remark, that this gentleman, in former fits of the gout, had suffered as much constitutional indisposition, when the parts affected were wrapped up in *flannel*, as under the *present mode of treatment.*

We have so frequently experienced the speedily beneficial effects of reduced temperature in the cure of gouty inflammation, that we cannot subscribe our assent to the conclusion of Mr. Edlin,* that all the baneful consequences in Mr. Baker's case are to be imputed to the use of that remedy. One in particular, the *fainting fit*, (which, whenever it happens, must excite the greatest alarm,) we see no reason to attribute to the use of cold water: for we have met with an instance of its occurrence to a most alarming degree, in a case which has come under our care, since writing the above statement, and where *no cold* whatever had been applied. We truly lament with Mr. Edlin the unfortunate issue of Mr. Baker's case; which, after all, however, is but a solitary instance; for as to that of Mr. Owen, it is, according to his own confession, a *mere old woman's story.*

We remain, &c.

JAMES SCOTT,

*Bromley, Kent*, Dec. 10, 1804. R. T. TAYNTON.

---

* Vide Mr. Edlin's Pamphlet.

